# Golden spiral or Fibonacci spiral: Which is more beautiful and why?

**DOI:** 10.1177/20416695241243319

**Published:** 2024-04-09

**Authors:** Ronald Hübner

**Affiliations:** Department of Psychology, 26567 University of Konstanz, Konstanz, Germany

**Keywords:** golden spiral, Fibonacci spiral, Archimedean spiral, beauty, curvature, fair curve

## Abstract

Spirals, with their widespread presence in both nature and culture, are universally admired. Although there are different types, such as the Archimedean, logarithmic, and golden spirals, they are indiscriminately considered as beautiful. This universal view might explain the lack of studies investigating aesthetic differences among spirals. To show that there are indeed differences, the beauty of the golden spiral was compared with that of the Fibonacci spiral in this empirical study. Since the curvature of the golden spiral changes continuously, whereas that of the Fibonacci spiral does so in steps, the golden spiral was predicted to be aesthetically preferred. The results clearly confirmed this prediction. That the difference in preference was really based on the continuity versus discontinuity of the curvature is supported by the further result that an Archimedean spiral was preferred over a Dürer spiral, which similarly differed in their continuity.

Spirals are common both in nature and in culture. They were created by humans as early as 11,000 BC and appeared independently in different places. Spirals are not only important for mathematics and natural sciences but also play an important role as motifs in art and design. The British art critic and writer Theodore Andrea Cook (1867–1928), in his book “The Curves of Life” (Cook, 1914), proposed that the spiral, with its open-ended curve, gives a sensation of continuous motion. He suggested that the obvious beauty of spirals may be due to their association with the fundamental processes of life and growth. Indeed, although various types of spirals have been identified or invented (Tsuji & Müller, 2019), spirals are indiscriminately regarded as beautiful. Nevertheless, it seems reasonable to assume that they also differ in their beauty. Unfortunately, there is a lack of systematic empirical studies on this topic, at least as far as I know. This study is a first step towards closing this gap.

Often, scientists, but also artists, admire specific spirals not only because of their form, but also because of the fascinating mathematics behind them. A prominent example is the logarithmic spiral, the favorite spiral of the Swiss Mathematician Jakob I Bernoulli (1655–1705). Bernoulli was so fascinated by logarithmic spirals that he wanted to have one engraved on his gravestone. After his death, however, for whatever reason, an Archimedean spiral was engraved instead of a logarithmic one (Livio, 2008).

Looking at the description and evaluation of spirals in the literature, the so-called *golden spiral*, a special case of the logarithmic spiral, seems to be even more popular. This also holds for the Fibonacci Spiral, which is named after the Italian mathematician Leonardo Fibonacci (1170–1240), and closely related to the golden spiral. It is even considered an approximation of the golden spiral and is often used because it is relatively easy to construct. The beauty of the Fibonacci Spiral partly lies in its presumed ubiquity in nature. Indeed, allegedly it can be found in various natural phenomena such as the arrangement of leaves on a stem, the branching of trees, the flowering of an artichoke, the form of the Nautilus, etc. (Tsuji & Müller, 2019).

In this study, I have investigated whether the golden spiral or the Fibonacci spiral is more beautiful. Fortunately, our knowledge about the beauty of curves allows us to predict which spiral should be perceived as more beautiful. To test the generalizability of the prediction that will be derived below, I have extended the comparison by also including the Archimedean spiral and its approximation proposed by Albrecht Dürer (1525) into the investigation, because the spirals in this pair differ in a similar way as those in the other pair. But before we make any predictions about the beauty of the different spirals, we first take a closer look at the characteristics of the spirals under consideration and at the beauty of curves.

## Types of Spirals

### Archimedean Spiral

The Archimedean spiral is a relatively simple spiral whose distance between the turns remains constant. Formally, its distance *ρ* (rho) from the center to one point on the spiral is proportional to the angle φ (phi) of rotation in radians. The equation of the (generalized) Archimedean spiral in the polar coordinate system is written as:

ρ=k+aφ.

**Figure 1.**
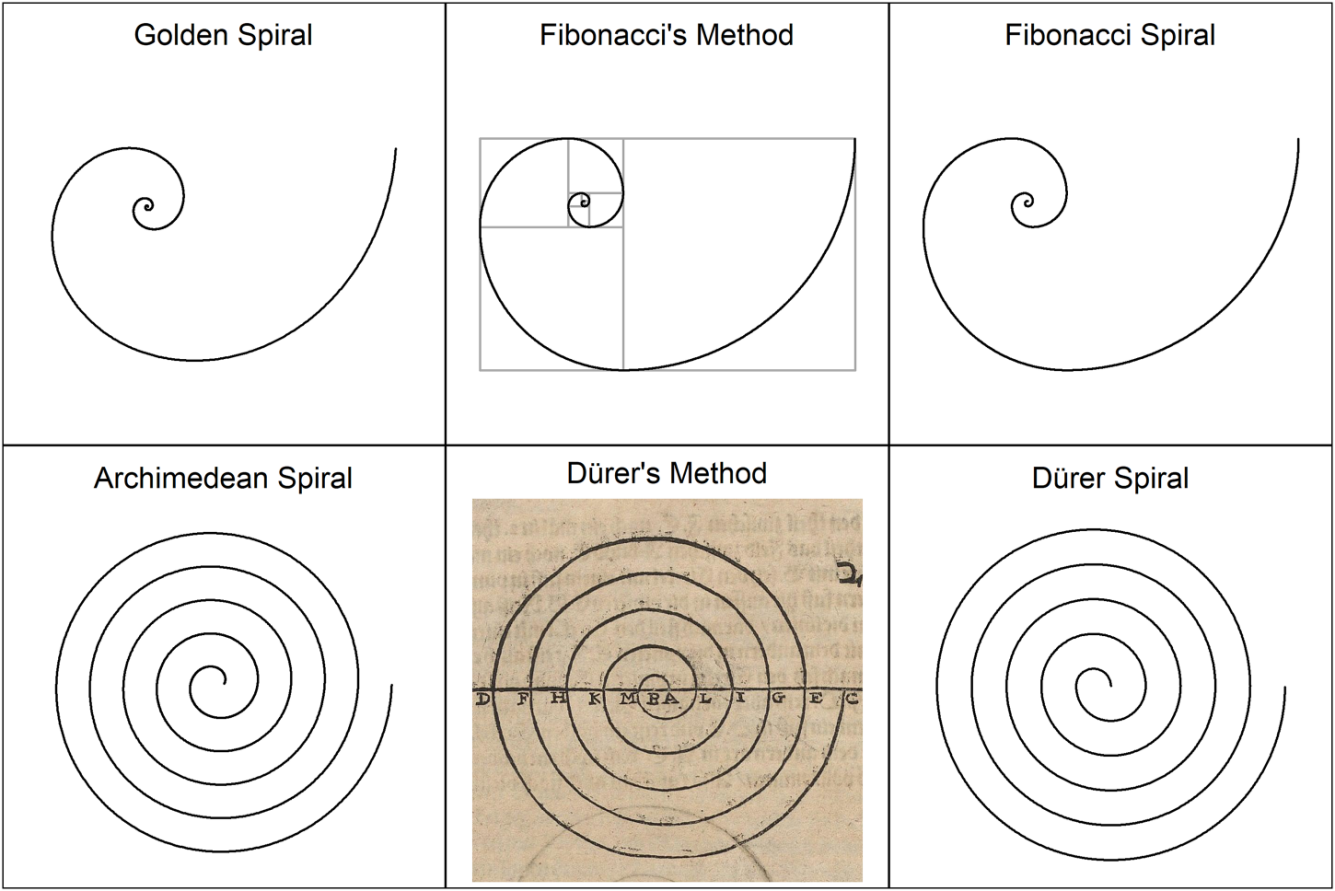
Different types of spirals.

*Note.* Upper row: The spiral on the left is an example of a golden spiral. The middle panel illustrates the method for constructing a corresponding Fibonacci spiral. The right panel shows the Fibonacci spiral approximating the golden spiral in the left panel. Lower row: The spiral on the left is an example of an Archimedean spiral. The middle panel shows a drawing by Albrecht Dürer (1525) demonstrating his method of approximating an Archimedean spiral by semicircles. The spiral on the right shows the approximation of the Archimedean spiral on the left using this method.

While the parameter *a* determines the distance between the turns, the parameter *k* moves the inner start point of the spiral outward from the origin. The usual Archimedean spiral is given if *k *= 0 (Polezhaev, 2019). For our objective, however, we need *k* > 0, because for such spirals Albrecht Dürer (1525) proposed an approximation by means of half circles with different radii and centers. An example of such an Archimedean spiral and its approximation is shown in the lower row in Figure 1.

### Logarithmic Spiral

The first known description of a logarithmic spiral was provided by Albrecht Dürer (1525). More than a century later, the curve was discussed by Descartes (1637/1985), and later extensively investigated by Jacob I Bernoulli, as mentioned. The logarithmic spiral differs from the Archimedean spiral in that the distances between the turns increase in geometric progression. A logarithmic spiral has two parameters *a* and *b*.

$$\rho =ae^{b\varphi },\;\;a>0.$$

Parameter *b* is often called the *growth factor*. If *b *= 0.3063, then the spiral is called the *golden spiral* (Polezhaev, 2019). The reason is that it gets wider (or further from its origin) by a factor that is the *golden ratio*, for every quarter turn it makes. An example of a golden spiral can be seen in the upper row of Figure 1. Dürer (1525) also proposed an approximation of the golden spiral. He suggested connecting quarter circles.

### Fibonacci Spiral

Similar to Dürer's (1525) idea, but a much more respected approximation of the golden spiral is the Fibonacci spiral, which is a graphical representation of the Fibonacci sequence (1, 1, 2, 3, 5, …), where each number is the sum of the two preceding ones. The Fibonacci spiral starts with a rectangle divided into 2 squares. In each step, a square the length of the longest side of the rectangle is added to the rectangle. The corners of these squares are then connected by quarter circles (for an example see Figure 1). Since the ratio between successive Fibonacci numbers approaches the golden ratio, the spiral becomes more similar to the golden spiral as more squares are added.

The Fibonacci spiral approximates the golden spiral in the similar way that the Dürer spiral approximates the Archimedean spiral, with the difference that quarter circles are connected instead of semicircles.

## Curvature and Beauty

**Figure 2.**
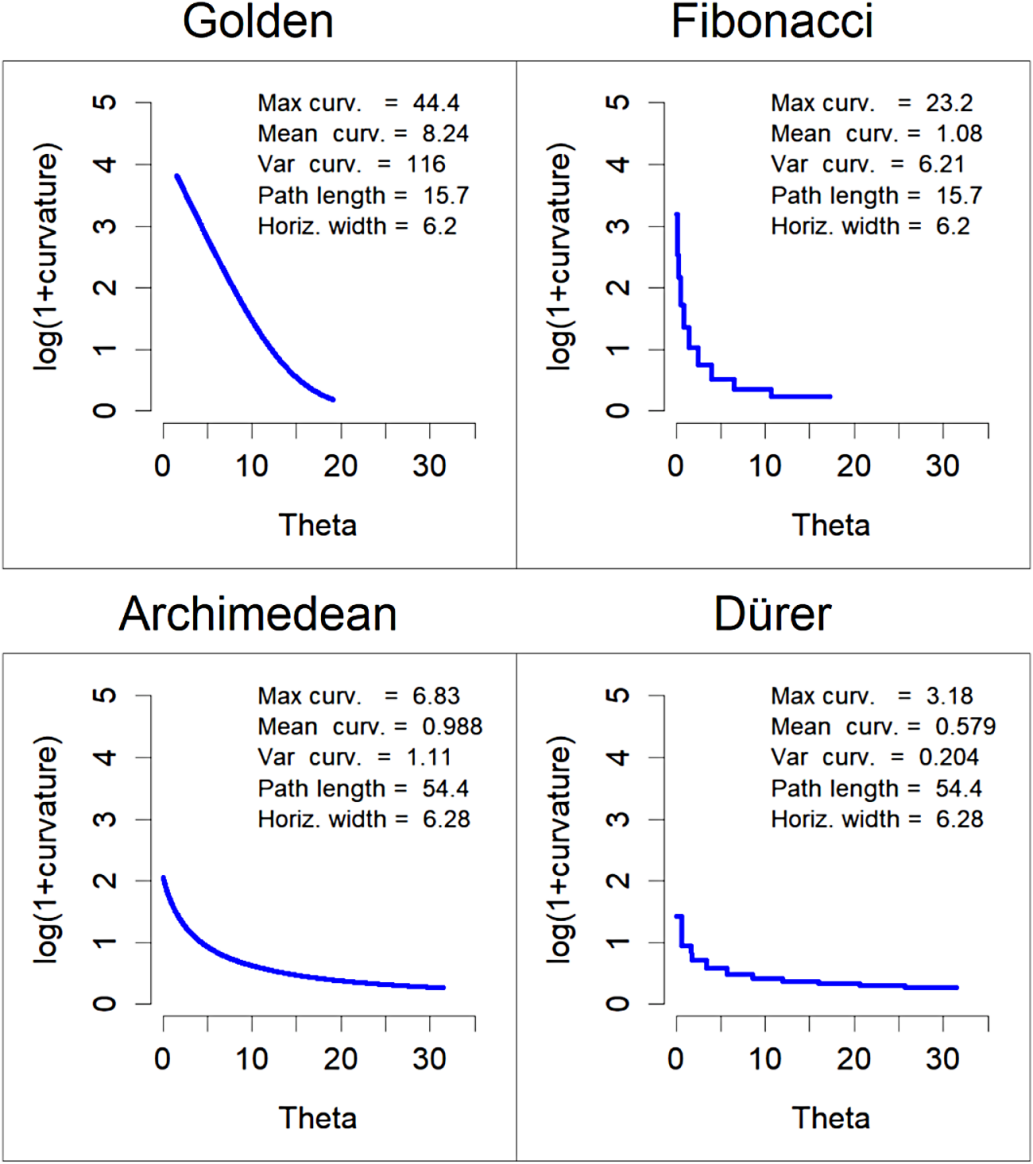
Curvature plots of the four spirals used as stimuli.

*Note*. In the legends various further features of the spirals are given (“curv.” stands for “curvature”).

The aesthetics of curves have been intensively researched in the field of design (Crăciun et al., 2020). To find the most beautiful curves, some designers examined the shapes of various natural and artificial objects (Harada et al., 1999; Harada & Yoshimoto, 2003). A common method to evaluate curves is to consider their curvature plots, that is, how the curvature changes along their path. In Figure 2 the curvature plots of the two left and two right spirals in Figure 1 are shown. As can be seen, for the golden spiral and the Archimedean spiral the curvature decreases continuously. In contrast, for the Fibonacci spiral and Dürer spiral the curvature remains constant along each circle segment (half or quarter circle) but changes abruptly from one to the next segment.

It is now widely accepted among researchers and designers that a curve is beautiful or, as they call it, *fair*, if its curvature does not change abruptly (Birkhoff, 1933; Farin & Sapidis, 1989; Kanaya et al., 2003). Many spirals meet this requirement. Even more, any curve with a monotone curvature of constant sign is deﬁned as a spiral (Gobithaasan & Miura, 2014). However, the reverse does not hold, that is, not all spirals have a monotone curvature. Famous examples are the two approximations, Fibonacci spiral and Dürer spiral, considered here. As we have seen in Figure 2, their curvature does not change continuously.

In this experiment I wanted to examine to what extent the assumptions about the beauty or fairness of curves can be used to predict the beauty of spirals. If we look at Figure 1, then the differences in curvature between the original spirals (golden, and Archimedean) and their approximations (Fibonacci, and Dürer) are hardly visible, especially for the golden spiral and the Fibonacci spiral. Nevertheless, they are present and, according to the idea of fairness of curves, should have an effect on their beauty. Accordingly, it can be hypothesized that the golden and the Archimedean spirals are preferred to their approximations. This hypothesis was tested in an experiment.

## Method

### Participants

One hundred and six participants (mean age 31.4 years, range = 18–71, SD = 9.11, 46 male) were recruited via social media for participation in the online study. The data collection also comprised other parts, whose results will be reported elsewhere. For completing the study, which altogether lasted about ten minutes, the participants received a voucher worth € 2. The experiment was conducted in accordance with the ethical guidelines of the University of Konstanz and the Declaration of Helsinki (1964) and its later amendments. Participants were informed of their right to quit the study at any time without reprisal and their informed consent was obtained by check-marking a box before the actual experiment started.

### Stimuli

Stimuli were four different spirals, a golden spiral, an Archimedean spiral, and their approximations, that is, a Fibonacci spiral and a Dürer spiral. All four are shown in Figure 1. The Archimedean spiral has five turns, and its parameters are *a *= 0.132, and *k *= *a*·π. The corresponding Dürer spiral also has five turns (10 half circles). The golden spiral has 3.038 turns, and its parameters are *a *= 0.0133, *b *= 0.306. The corresponding Fibonacci spiral consists of 11 quarter circles, which give 2.75 turns. The numbers of the corresponding Fibonacci sequence are: 1, 1, 2, 3, 5, 8, 13, 21, 34, 55, and 89. The curvature plot for these four spirals are shown in Figure 2. As can be seen, the original spirals and their approximations differ largely in the continuity of their curvature. What can also be seen, the spirals in each pair also differ with respect to other features such as maximum and mean curvature, and variance of curvature. However, the differences are not so dramatic that one would predict substantial differences in beauty.

### Procedure

At the beginning of the experiment, participants were shortly introduced to the topic and procedure. After consent and providing personal information (gender, age), a specific instruction for the required task was presented. To achieve a high quality of stimuli presentation, the participants were informed that they had to use a computer. The program stopped if a mobile device was used. All stimuli were displayed in black on a white rectangle of 1000 × 500 pixels on a gray background on the screen. The task was to decide which of the two adjacent spirals they liked best. The two pairs were Golden-Fibonacci and Archimedean-Dürer. Each pair occurred in a left-right and right-left spiral-type order. The corresponding four trials were presented in a randomized order for each participant.

## Results

**Figure 3.**
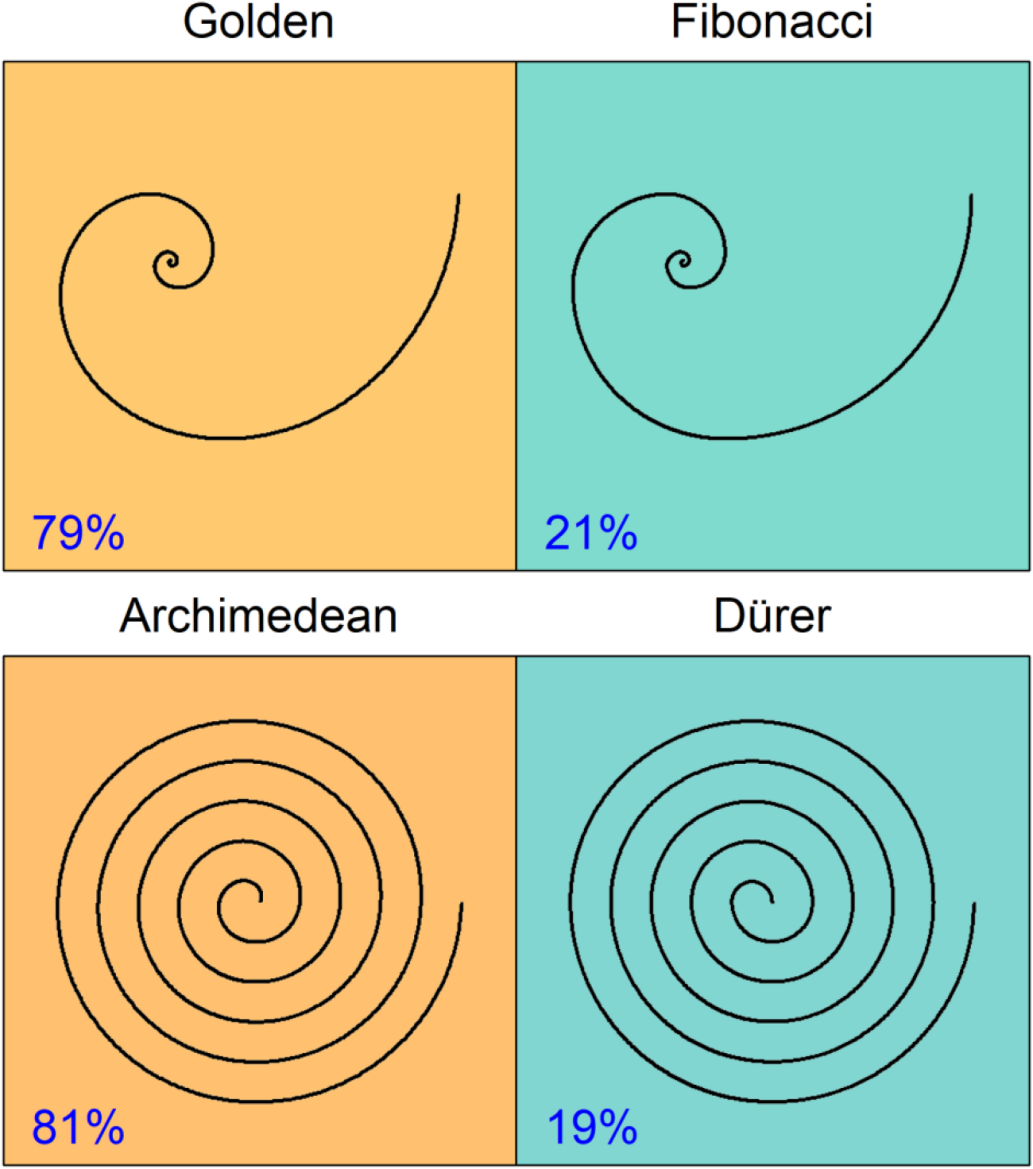
Results of the preference between the two pair of spirals.

*Note*. The percentages of the choices within each pair are shown in the respective bottom left corner. They are also represented by the background color of the panels, where the range of the heat map goes from 1 (blue) to 100 (red). It should be noted that the percentages can only be compared within each pair.

The results are so clear that no inferential statistics are required to interpret them. As predicted, the original spirals were chosen much more frequently than their approximations. While 79.2% of the participants chose the golden spiral, only 20.8% selected the Fibonacci spiral. At 81.1% compared to 18.9%, the preference of the Archimedean spiral over the Dürer spiral was very similar. The choice percentages are again shown in Figure 3 together with the corresponding stimuli.

## Discussion

The aim of this study was to demonstrate that spirals, which are usually considered beautiful regardless of their type, nevertheless differ in their aesthetics. For this purpose, the golden spiral and the Fibonacci spiral were chosen as a famous pair for comparison. Although the Fibonacci spiral is perceptually very similar to the golden spiral and is therefore regarded as its approximation, the two spirals differ in one important qualitative characteristic. While the curvature of the golden spiral changes continuously, that of the Fibonacci spiral varies discontinuously. Because discontinuities in curvature are usually not liked, it was expected that the Fibonacci spiral is perceived as less beautiful than the golden spiral. This is exactly what was found. Around 80% of the participant preferred the golden spiral over the Fibonacci spiral. That the lower preference for the Fibonacci spiral is indeed due to the discontinuities in its curvature is supported by the other results of this study obtained with the Archimedean and the Dürer spiral, a pair that shows similar differences between their curvatures. It should be noticed that these findings also indicate our high sensitivity to discontinuous changes in curvature.

In particular, the result of the Fibonacci spiral may come as a surprise, as it is especially fascinating. It is not only seen as an approximation of the golden spiral but is also considered rather beautiful in its own right. The present results, however, suggest that the beauty of the Fibonacci spiral may be overrated, probably due to its close relation with the golden ratio and its associated beauty myth (Boselie, 1992; Höge, 1997; Markowsky, 1992).

### Limits and Future Directions

This small study clearly has its limitations. First of all, I have only considered whether changes in curvature are abrupt or continuous as the cause of the differences in beauty between the original spirals and their approximations. However, despite its high predictive power, there may be alternative or additional causes. For instance, the original spirals and their approximation also differ in whether there are sections with constant curvature or not. As such sections only occur in the approximations, this difference could also account for the present results. Unfortunately, there is little evidence for this idea so far. The only clue I know is from Martin (1906). In her study, she had various geometric forms assessed according to their beauty. She found that “[t]he arcs of the circles seem to be least liked …” (p. 151). However, as long as this result has not been replicated with modern methods, the effect of constant curvature on the beauty of lines remains open.

A further limitation due to the rather specific research question of the present study is that only differences in beauty between originals and their approximations were examined, but not between the originals. In view of the results, however, it seems promising to compare the two originals as well as other types of spirals. Even if all these spirals have a constantly changing curvature, it is likely that at least some of them will be judged differently beautiful. In this case, differences in other features such as symmetry (Bertamini et al., 2013; Fitousi & Algom, 2024) or balance (Hübner & Fillinger, 2016; Hübner & Thömmes, 2019) could be responsible. It is the task of future studies to investigate whether this is the case.
